# Association between fibroblast growth factor 23 and coronary artery calcification in patients with chronic kidney disease: a meta-analysis

**DOI:** 10.3389/fendo.2026.1900670

**Published:** 2026-09-07

**Authors:** Yang Yi, Yingdan Zhao, Hanqing Wang, Xinxin Jiang, Yi Xuan

**Affiliations:** 1 Department of Nephrology, Jing’an District Center Hospital of Shanghai, Fudan University, Shanghai, China; 2 Department of Nephrology, Jiading Branch of Shanghai General Hospital, Shanghai Jiao Tong University School of Medicine, Shanghai, China

**Keywords:** chronic kidney disease, coronary artery calcification, fibroblast growth factor 23, hemodialysis, meta-analysis

## Abstract

**Background:**

Coronary artery calcification (CAC) is a common cardiovascular complication in patients with chronic kidney disease (CKD). Fibroblast growth factor 23 (FGF23), a key regulator of phosphate metabolism, has been implicated in vascular calcification, but studies evaluating its association with CAC in CKD have yielded inconsistent findings. This meta-analysis aimed to evaluate the association between circulating FGF23 levels and CAC in patients with CKD.

**Methods:**

PubMed, Embase, Web of Science, Wanfang, and CNKI were systematically searched for observational studies evaluating the association between circulating FGF23 levels and CAC in adult patients with CKD were included. Odds ratios (ORs) and 95% confidence intervals (CIs) were pooled using random-effects models accounting for the influence of potential heterogeneity.

**Results:**

Twelve cross-sectional studies involving 3, 466 patients with CKD were included. Elevated circulating FGF23 levels were significantly associated with a higher prevalence of CAC (OR: 2.11, 95% CI: 1.70–2.63, *p* < 0.001), with no significant heterogeneity (I² = 0%). Sensitivity analyses confirmed the robustness of the findings (ORs: 2.05–2.24). Consistent associations were observed across subgroups according to age (<56 vs. ≥56 years; OR: 1.84 vs. 2.32), sex distribution (<60% vs. ≥60% men; OR: 2.41 vs. 1.82), diabetes prevalence (<30% vs. ≥30%; OR: 2.17 vs. 2.05), dialysis status (dialysis vs. predialysis CKD; OR: 2.15 vs. 2.01), FGF23 assay type (cFGF23 vs. iFGF23; OR: 2.44 vs. 2.03), CAC severity, and study quality (all *p* for subgroup difference >0.05). Meta-regression analyses showed that study-level characteristics did not significantly influence the association.

**Conclusions:**

Elevated circulating FGF23 levels are associated with a higher prevalence of CAC in patients with CKD. These findings suggest that elevated FGF23 levels may serve as a potential biomarker associated with vascular calcification in CKD, although prospective studies are needed to clarify causality and clinical utility.

This meta-analysis was conducted in accordance with established methodological recommendations, adhering to the PRISMA 2020 guidelines (32) and the Cochrane Handbook for Systematic Reviews of Interventions (33) throughout protocol development, literature screening, data extraction, statistical synthesis, and interpretation of the findings.

**Systematic review registration:**

https://www.crd.york.ac.uk/prospero/, identifier CRD420261415908.

## Introduction

Chronic kidney disease (CKD) is a major global health problem affecting approximately 10–15% of the adult population worldwide and is associated with substantial morbidity, mortality, and healthcare expenditure (1, 2). As kidney function progressively declines, patients with CKD are exposed to a markedly increased risk of cardiovascular disease (CVD), which remains the leading cause of death in this population (3, 4). Among the various cardiovascular abnormalities observed in CKD, coronary artery calcification (CAC) is particularly common and clinically important (5, 6). The prevalence and severity of CAC increase progressively with worsening kidney function and are especially high among patients with end-stage renal disease (ESRD) receiving dialysis (7). Extensive evidence has demonstrated that CAC is associated with increased risks of myocardial infarction, heart failure, cardiovascular mortality, and all-cause mortality in patients with CKD (8, 9). The pathogenesis of CAC in CKD is multifactorial and involves both traditional cardiovascular risk factors, such as advanced age, diabetes, hypertension, smoking, and dyslipidemia, and nontraditional CKD-related factors, including chronic inflammation, oxidative stress, endothelial dysfunction, and disturbances in mineral and bone metabolism (10, 11). Consequently, identifying biomarkers that can reflect or predict the development of CAC may facilitate risk stratification and improve cardiovascular management in this high-risk population.

Fibroblast growth factor 23 (FGF23) is a phosphaturic hormone secreted primarily by osteocytes and osteoblasts and serves as a key regulator of phosphate and vitamin D homeostasis (12, 13). Physiologically, FGF23 promotes renal phosphate excretion and suppresses the synthesis of active vitamin D, thereby maintaining mineral balance (12). Circulating FGF23 can be measured as intact FGF23 (iFGF23), which represents the biologically active hormone, or as C-terminal FGF23 (cFGF23), which reflects both intact hormone and cleavage fragments (13). As renal function deteriorates, circulating FGF23 concentrations rise substantially and often increase before overt abnormalities in serum phosphate become apparent, making FGF23 one of the earliest biomarkers of CKD-mineral and bone disorder (CKD-MBD) (14, 15). Beyond its role in mineral metabolism, elevated FGF23 levels have been linked to left ventricular hypertrophy, heart failure, cardiovascular events, CKD progression, and increased mortality, suggesting that FGF23 may contribute directly to cardiovascular injury (16, 17). Mechanistically, elevated FGF23 levels may promote vascular calcification by reflecting phosphate retention and disordered mineral metabolism, while also interacting with pathways related to inflammation, oxidative stress, endothelial dysfunction, and osteogenic transformation of vascular smooth muscle cells (VSMCs) (18, 19). Over the past decade, several observational studies have investigated the association between circulating FGF23 levels and CAC in patients with CKD, but the findings have been inconsistent, likely due to differences in patient populations, CKD severity, dialysis status, FGF23 assays, and CAC definitions (20–31). Therefore, we performed this systematic review and meta-analysis to comprehensively evaluate the association between circulating FGF23 levels and CAC in patients with CKD and to determine whether this relationship is consistent across clinically relevant subgroups.

## Methods

This meta-analysis was conducted in accordance with established methodological recommendations, adhering to the PRISMA 2020 guidelines (32) and the Cochrane Handbook for Systematic Reviews of Interventions (33) throughout protocol development, literature screening, data extraction, statistical synthesis, and interpretation of the findings. The review protocol was prospectively registered in the PROSPERO database (registration No. CRD420261415908).

### Literature search

A comprehensive literature search was performed in PubMed, Embase, Web of Science, Wanfang, and China National Knowledge Infrastructure (CNKI) to identify eligible studies. The search strategy combined terms of (“fibroblast growth factor 23” OR FGF23 OR “FGF-23” OR “FGF 23”) AND “coronary” AND “calcification” AND (“chronic kidney disease” OR CKD OR “chronic renal insufficiency” OR “chronic renal failure” OR “renal insufficiency” OR “kidney failure” OR “end-stage renal disease” OR ESRD OR “dialysis” OR hemodialysis OR haemodialysis OR “peritoneal dialysis”). Only full-text studies published in English or Chinese and conducted in human populations were considered eligible. In addition, the reference lists of relevant original articles and review papers were manually screened to identify any additional potentially relevant studies. The literature search covered all databases from inception through March 18, 2026. Detailed search strategies for each database are provided in **Supplementary File 1**.

### Criteria of study selection

Study eligibility was determined according to the PICOS framework.

P (Population): Adult patients (≥ 18 years) with CKD were eligible for inclusion. CKD diagnosis was required to be based on established clinical criteria, including CKD stage defined by estimated glomerular filtration rate (eGFR), ESRD, or maintenance dialysis status, as reported in the original studies.

I/E (Exposure): The exposure of interest was elevated circulating FGF23 levels measured in serum or plasma. Both iFGF23 and cFGF23 assays were eligible. Studies evaluating FGF23 as a categorical variable, such as quartiles, tertiles, median-based categories, predefined cutoffs, or highest versus lowest categories, were included. The cutoffs for defining a high blood level of FGF23 were consistent with those used in the original studies.

C (Comparator): The comparator consisted of participants with lower circulating FGF23 levels, as defined by the reference group in each study.

O (Outcome): The outcome of interest was CAC, assessed using computed tomography (CT), electron-beam CT, multidetector CT, or other validated imaging modalities. Eligible studies evaluated the presence of CAC as a binary outcome (CAC versus no CAC) or CAC severity. In general, CAC was assessed using computed tomography-derived coronary artery calcium scores (CACS), primarily based on the Agatston scoring system (34). Because the included studies used different CACS thresholds, CAC outcomes were categorized according to calcification severity. Overall CAC was defined as the presence of coronary calcification, corresponding to CACS > 0 or > 10 according to the original study definitions. Moderate-to-severe CAC was defined as CACS ≥ 100, whereas severe CAC was defined as CACS ≥ 400 (34).

S (Study Design): Observational studies, including prospective cohort studies, retrospective cohort studies, case–control studies, and cross-sectional studies, were eligible for inclusion. Only full-length articles published in peer-reviewed journals were considered. Studies were required to provide original data regarding the association between circulating FGF23 levels and CAC in patients with CKD.

Exclusion criteria: Studies were excluded if they: (1) included pediatric populations (< 18 years), non-CKD populations, or focusing on kidney transplant recipients; (2) did not evaluate circulating FGF23 levels as the exposure of interest; (3) did not assess CAC or failed to provide data regarding the association between FGF23 and CAC; (4) reported vascular calcification outcomes without separately presenting CAC data; (5) were reviews, meta-analyses, editorials, letters, case reports, animal studies, *in vitro* studies, or narrative reviews; (6) lacked sufficient data to calculate effect estimates; (7) represented duplicate publications or analyses of overlapping populations, in which case the study with the largest sample size was retained; or (8) were not available as full-text articles.

### Assessment of study quality

The methodological quality of the included studies was independently assessed by two reviewers using a modified Newcastle–Ottawa Scale (NOS) adapted for cross-sectional studies (35, 36). The modified NOS evaluates study quality across three domains: selection (representativeness of the sample, clearly defined CKD population, non-respondents, and ascertainment of exposure; maximum 4 stars), comparability (adjustment for age and other important confounders; maximum 2 stars), and outcome assessment (use of validated coronary artery calcification assessment, outcome assessment, and appropriate statistical analysis; maximum 3 stars). Studies with higher scores were considered to have better methodological quality. Any disagreements were resolved through discussion until consensus was reached.

### Extraction of study data

Data extraction was independently completed by two reviewers using a standardized and pilot-tested extraction form. The collected information included study-related data (first author, publication year, country, and study design), patient characteristics (sample size, stage of CKD, mean age, sex distribution, proportion of diabetic patients, and proportion of patients on dialysis), exposure-related variables (methods for measuring circulating FGF23 and cutoffs used for defining a high blood FGF23 level), methods for evaluating CAC, number of patients with CAC, and covariates adjusted for in the analyses evaluating the association between circulating FGF23 and CAC in patients with CKD.

### Statistical analyses

The relationship between circulating FGF23 level and CAC in patients with CKD was quantified by pooling odds ratios (ORs) and corresponding 95% confidence intervals (CIs) comparing patients with elevated versus lower circulating FGF23 levels (33). When multiple adjusted models were reported, the estimates derived from the model with the most comprehensive adjustment were selected for analysis. If required, effect sizes and standard errors were calculated from the available 95% CIs or *p* values. Prior to data synthesis, all effect estimates were transformed to the natural logarithmic scale to improve distribution normality and stabilize variances (33).

Between-study heterogeneity was assessed using the Cochrane Q test and the I² statistic (37). Heterogeneity was classified as low (I² < 25%), moderate (I² = 25–75%), or high (I² > 75%). The pooled estimates were generated using the inverse-variance method under a random-effects model based on the DerSimonian–Laird approach to account for potential inter-study variability (33). Robustness of the pooled findings was evaluated through leave-one-out sensitivity analyses, in which individual studies were sequentially omitted (38).

Predefined subgroup analyses were conducted according to the mean age of the patients, sex distribution, proportion of diabetic patients, dialysis status, type of FGF23 assays (cFGF23 vs. iFGF23), cutoffs of CACS, and quality scores. Medians of continuous variables were used as the cutoff values for defining subgroups to maintain balanced number of studies in each subgroup. In addition, univariate meta-regression analyses were performed to examine whether study-level factors, including sample size, mean age, male proportion, proportion of patients with diabetes, cutoffs of CACS, and NOS score, influenced the association between circulating FGF23 and CAC of patients with CKD (33).

Potential publication bias was evaluated by visually inspecting funnel plot symmetry and further assessed using Egger’s regression test (39). A two-sided *p* value < 0.05 was considered statistically significant. Statistical analyses were performed using RevMan version 5.3 (Cochrane Collaboration, Oxford, UK) and Stata version 17.0 (StataCorp, College Station, TX, USA).

## Results

### Literature search results

**Figure 1:**
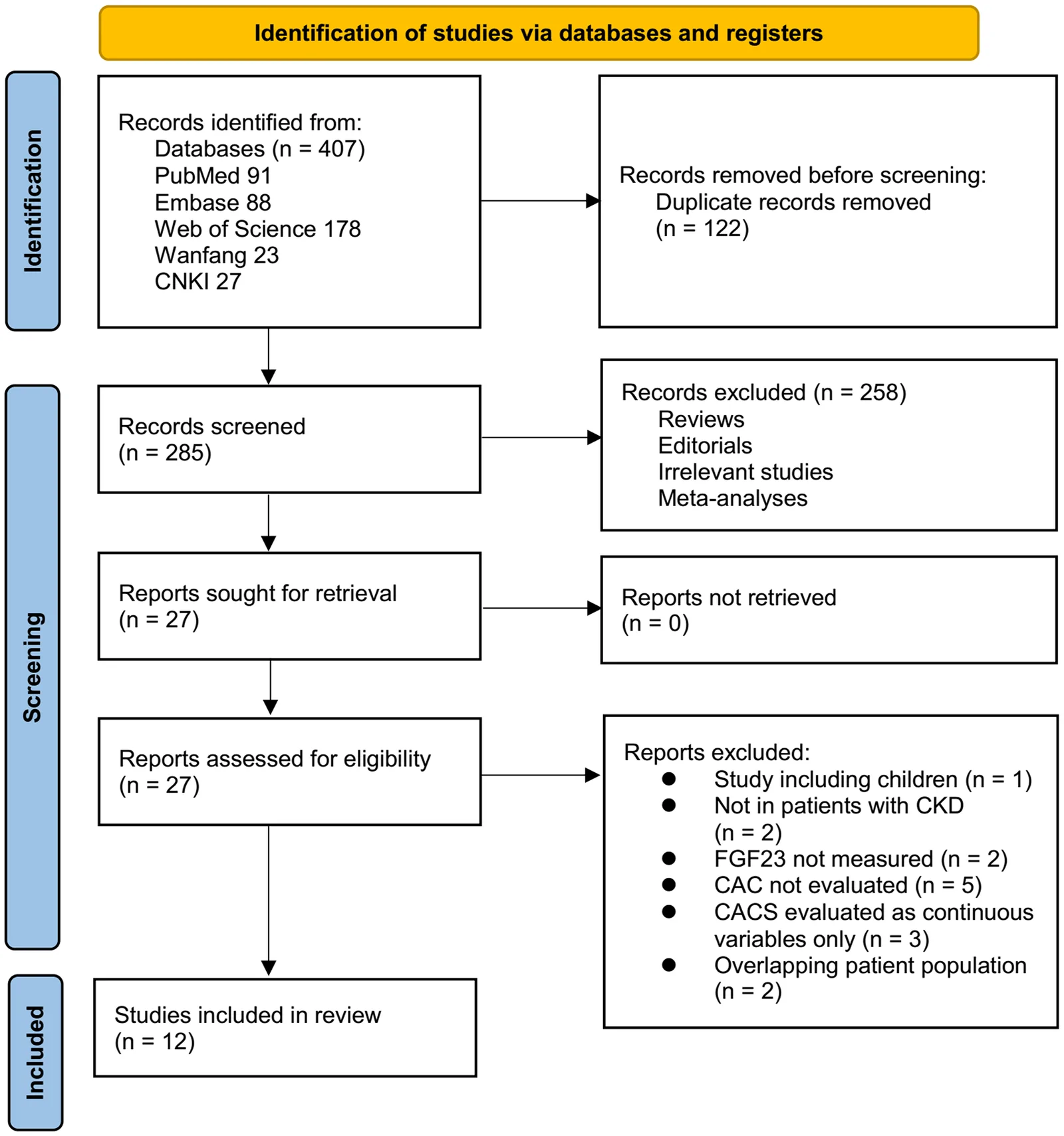
Flow diagram of the study selection process.

The process of study identification and selection is presented in **Figure 1**. A total of 407 records were initially identified through searches of the five databases, and 122 duplicate records were removed. After screening titles and abstracts, 258 articles were excluded because they did not satisfy the predefined eligibility criteria. Subsequently, the full texts of 27 potentially relevant articles were reviewed independently by two investigators, of which 15 were excluded for the reasons shown in **Figure 1**. Ultimately, 12 eligible studies were included in the final quantitative synthesis (20–31).

### Summary of study characteristics

The main characteristics of the included studies are summarized in **Table 1**. A total of 12 cross-sectional studies published between 2009 and 2025 were included (20–31), comprising 3, 466 patients with CKD. The studies were conducted in diverse geographic regions, including China (22–24, 26, 28, 30, 31), the United States (20, 29), France (21), Turkey (25), and South Korea (27). The included populations covered a broad spectrum of kidney disease severity, including predialysis CKD stages 1–5, ESRD, and patients receiving maintenance hemodialysis or peritoneal dialysis. Four studies were conducted exclusively in maintenance hemodialysis populations (22, 23, 25, 29), whereas others enrolled predialysis CKD patients (20, 21, 27, 28, 30, 31) or mixed cohorts of dialysis and non-dialysis patients (24, 26). The mean age of participants ranged from 40.2 to 70.8 years, and the proportion of men ranged from 51.0% to 66.4%, indicating a predominantly middle-aged to elderly population with a slight male predominance. The prevalence of diabetes varied substantially across studies, ranging from 0% to 58.0%. All studies measured circulating FGF23 using enzyme-linked immunosorbent assay (ELISA) methods. Seven studies evaluated iFGF23 (22–26, 28, 30), whereas five studies assessed cFGF23 (20, 21, 27, 29, 31). Eleven studies used serum samples (20–24, 26–31), while one study (25) measured FGF23 in plasma. Cutoffs to define high FGF23 levels were determined using median values (22, 23, 25, 26, 28–31) in eight studies, tertile comparisons in two studies (20, 21), and receiver operating characteristic (ROC) curve analysis in one study (24), and predefined thresholds (27) in another study. CAC was assessed using computed tomography-based techniques in all studies according to the Agatston scoring system. However, the definitions of CAC varied across studies, including CACS > 0 (23, 24, 29, 31), > 10 (30), ≥ 100 (20, 21, 26–28), and ≥ 400 (22, 25). Overall, 1287 patients had CAC. All studies reported adjusted effect estimates and controlled for age and other potential confounders, including sex, diabetes, kidney function, mineral metabolism parameters, inflammatory markers, cardiovascular risk factors, and dialysis-related variables, although the extent of adjustment differed among studies.

**Table 1:** Characteristics of the included studies.

Study	Country	Design	No. of patients included	CKD definition	Mean age (years)	Men (%)	DM (%)	Dialysis (%)	Methods for measuring FGF23	Cutoff of FGF23	CAC evaluation	No. of patients with CAC	Variables adjusted
Gutiérrez 2009 (20)	USA	CS	162	Pre-dialysis CKD (eGFR ≤60 ml/min/1.73m²), stages 3–4 (excluded stage 5 CKD)	61.8	65.5	41.4	0	Serum, cFGF23, ELISA	T3:T1, 150 RU/ml	Cardiac CT (64-slice), Agatston score, ≥100 units	100	Age, sex, race, eGFR, phosphate, BMI, DM, hypertension, vitamin D use, phosphorus binder use, CRP, BNP, and 1, 25(OH)_2_D
Morea 2012 (21)	France	CS	195	CKD Stages 1-5; eGFR 33.2 [6.5-91.9] mL/min/1.73m²; Stage 1-2 (9.7%), Stage 3 (46.7%), Stage 4-5 (43.6%)	70.8	57.4	32.3	0	Serum, cFGF23, ELISA	T3:T1, 173.3 RU/ml	Multi-detector CT (64-slice), Agatston score, ≥100 units	116	Age, sex, DM, and smoking
Wang 2014 (22)	China	CS	77	ESRD, maintenance hemodialysis (mean dialysis vintage 5.9 ± 4.4 years; range >15 months to ~12.5 years)	61.7	51.0	14.3	100	Serum, iFGF23, ELISA	Median, 370.7 pg/ml	256-detector-row CT, Agatston score, > 400 units	28	Age, sex, and SD-phosphorus
Zhang 2015 (24)	China	CS	150	CKD stages 3-5D (non-dialysis stages 3-5, plus PD and HD)	56.6	56.7	14.7	PD: 33.3 and HD: 26.7	Serum, iFGF23, ELISA	ROC analysis, 786.7 pg/ml	Multi-slice spiral CT, Agatston score, > 0 units	85	Age, smoking history, DM, dialysis vintage, FBG, and hs-CRP
Jia 2015 (23)	China	CS	182	ESRD, maintenance HD (≥ 3 months)	50.1	65.4	8.2	100	Serum, iFGF23, ELISA	Median, 5934.6 pg/ml	Spiral CT, Agatston score, > 0 units	52	Age
Turan 2016 (25)	Turkey	CS	229	ESRD, maintenance HD	58.7	51.0	24.0	100	Plasma, iFGF23, ELISA	Median, 53.5 pg/ml	Multi-slice CT (16-slice), ≥ 400 units	66	Age, sex, DM, time on dialysis, and cIMT
Yi 2018 (26)	China	CS	181	CKD stages 1–5 and maintenance HD	55.6	NR	16.9	HD: 42.0	Serum, iFGF23, ELISA	Median, 199.9 pg/ml	Multi-slice CT, Agatston score, ≥100 units	49	Age, eGFR, serum phosphorus (P), and calcium-phosphorus product (Ca×P)
Hyun 2019 (27)	South Korea	CS	1435	CKD stages 1-5 (predialysis); mean eGFR 54.2 ± 31.3 mL/min/1.73m²	53.6	59.9	36.5	0	Serum, cFGF23, ELISA	Predefined, 30.0 RU/ml	Multi-slice CT, Agatston score, ≥100 units	358	Age, sex, waist and hip ratio, systolic BP, DM, CVD, eGFR, LDL cholesterol, hemoglobin, albumin, hsCRP, urine PCR, current smoking, calcium, phosphorus, 25-hydroxyvitamin D, intact PTH, 24-h urine calcium, 24-h urine phosphate
Jiang 2019 (28)	China	CS	182	CKD stages 1-5 (stage defined according to K/DOQI classification)	40.2	57.1	0.0	0	Serum, iFGF23, ELISA	Median, 150.5 pg/ml	Multi-slice CT, Agatston score, ≥100 units	51	Age, eGFR, serum phosphorus (P), and calcium-phosphorus product (Ca×P)
Fitzpatrick 2020 (29)	USA	CS	391	ESRD, incident HD (initiating dialysis, 3-month average lab values from start)	55.0	60.0	58.0	100	Serum, cFGF23, ELISA	Median, 527.0 RU/ml	Spiral CT, Agatston score, > 0 units	195	Age, sex, race, CCI, BMI, systolic BP, albumin, CRP, serum phosphate, serum corrected calcium, serum klotho
Zhu 2023 (30)	China	CS	128	Non-dialysis CKD (Stages 1-5)	55.8	66.4	44.5	0	Serum, iFGF23, ELISA	Median, 25.2 ng/ml	Cardiac CT, Agatston score, > 10 units	58	Age, sex, diabetes, 25(OH)D3, phosphate, and eGFR
Peng 2025 (31)	China	CS	154	Non-dialysis CKD (Stages 2-5)	68.5	63.0	49.4	0	Serum, iFGF23, ELISA	Median, 127.3 ng/ml	CT, Agatston score, > 0 units	129	Age, BMI, CVD history, DM, eGFR, and Klotho

NR, not reported; CS, cross-sectional study; CKD, chronic kidney disease; eGFR, estimated glomerular filtration rate; ESRD, end-stage renal disease; DM, diabetes mellitus; FGF23, fibroblast growth factor 23; cFGF23, C-terminal fibroblast growth factor 23; iFGF23, intact fibroblast growth factor 23; ELISA, enzyme-linked immunosorbent assay; CAC, coronary artery calcification; CT, computed tomography; CACS, coronary artery calcium score; BMI, body mass index; CRP, C-reactive protein; BNP, B-type natriuretic peptide; 1, 25(OH)_2_D, 1, 25-dihydroxyvitamin D; SD, standard deviation; FBG, fasting blood glucose; hs-CRP, high-sensitivity C-reactive protein; cIMT, carotid intima-media thickness; HD, hemodialysis; BP, blood pressure; CVD, cardiovascular disease; LDL, low-density lipoprotein; PCR, protein-to-creatinine ratio; PTH, parathyroid hormone; Ca×P, calcium-phosphorus product; CCI, Charlson Comorbidity Index; 25(OH)D3, 25-hydroxyvitamin D3; ROC, receiver operating characteristic; RU, relative unit; T1, first tertile; T3, third tertile.

### Study quality assessment

Using the modified NOS for cross-sectional studies, the methodological quality of the included studies ranged from 6 to 9 stars (**Table 2**), which indicates generally moderate to high methodological quality. Overall, all studies enrolled clearly defined CKD populations, appropriately ascertained FGF23 exposure and CAC outcomes, and used suitable statistical analyses, although some studies lacked representative sampling, adjustment for multiple confounders, or information regarding non-respondents.

**Table 2:** Methodological quality assessment of the included studies using the modified Newcastle–Ottawa Scale for cross-sectional studies.

Study	Representativeness of the sample	Clearly defined CKD population	Non-respondents	Ascertainment of exposure	Adjustment for age	Adjustment for other confounders	Use of validate CAC evaluation score	Outcome assessment	Statistical test	Total
Gutiérrez 2009 (20)	1	1	0	1	1	1	1	1	1	8
Morea 2012 (21)	0	1	0	1	1	1	1	1	1	7
Wang 2014 (22)	1	1	1	1	1	1	1	1	1	9
Zhang 2015 (24)	0	1	0	1	1	1	1	1	1	7
Jia 2015 (23)	0	1	0	1	1	0	1	1	1	6
Turan 2016 (25)	1	1	0	1	1	1	1	1	1	8
Yi 2018 (26)	1	1	0	1	1	1	1	1	1	8
Hyun 2019 (27)	1	1	1	1	1	1	1	1	1	9
Jiang 2019 (28)	0	1	0	1	1	1	1	1	1	7
Fitzpatrick 2020 (29)	1	1	1	1	1	1	1	1	1	9
Zhu 2023 (30)	0	1	0	1	1	1	1	1	1	7
Peng 2025 (31)	0	1	1	1	1	1	1	1	1	8

Selection: representativeness of the sample, clearly defined CKD population, non-respondents, and ascertainment of exposure (maximum 4 stars). Comparability: adjustment for age and other important confounders (maximum 2 stars). Outcome: use of validated CAC assessment, outcome assessment, and appropriate statistical analysis (maximum 3 stars). Higher scores indicate better methodological quality.

### Results of the meta-analysis

**Figure 2:**
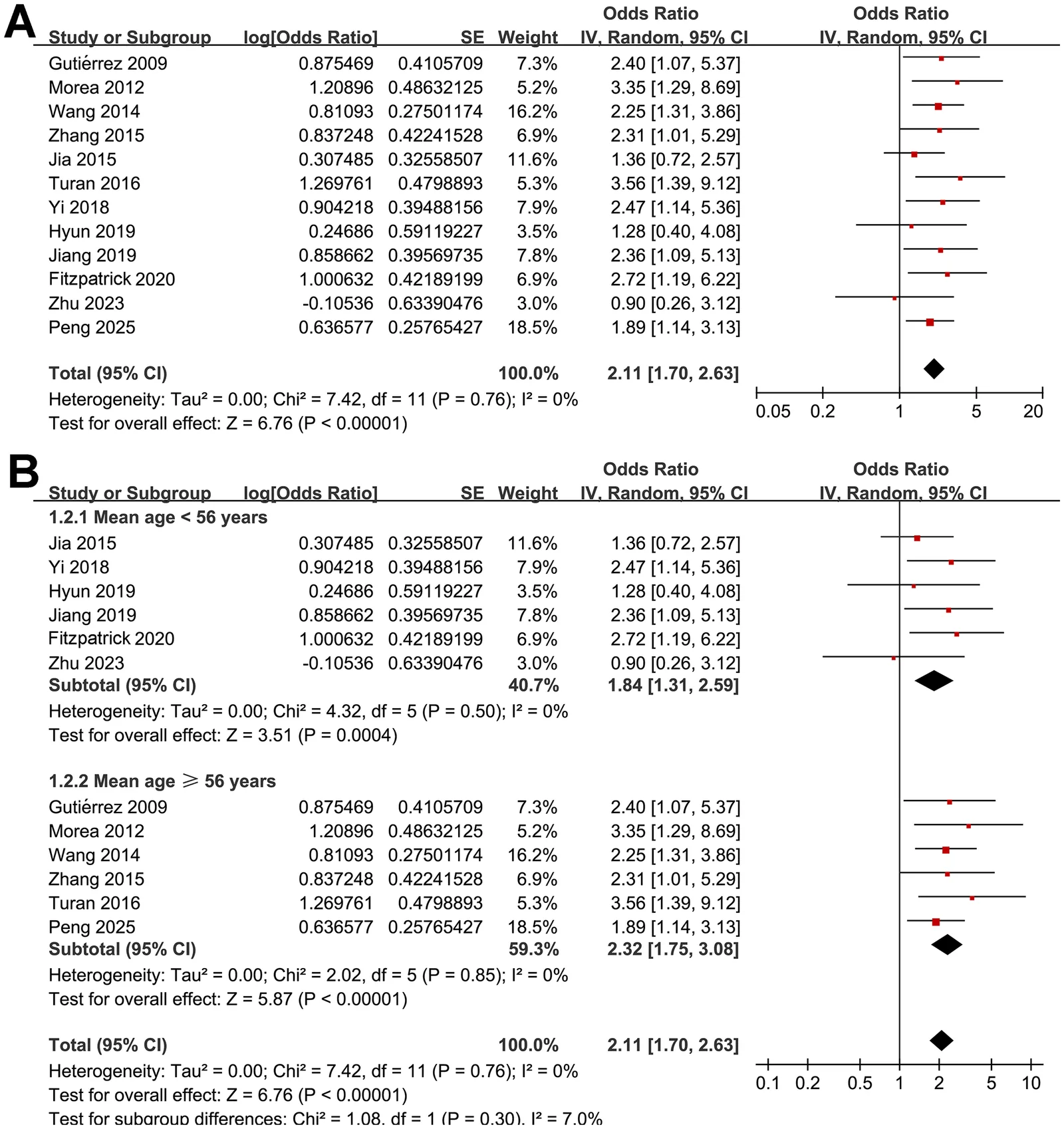
Forest plots showing the meta-analysis of the association between blood FGF23 level and CAC in adult patients with CKD: **(A)** forest plots for the overall meta-analysis; and **(B)** forest plots for the subgroup analysis according to the mean age of the patients.

The pooled analysis of the 12 studies (20–31) showed that elevated circulating level of FGF23 in CKD patients was significantly associated with a higher prevalence of CAC (OR: 2.11, 95% CI: 1.70–2.63, *p* < 0.001; **Figure 2A**). Between-study heterogeneity was low (Cochrane Q test *p* = 0.76; I² = 0%). In addition, leave-one-out sensitivity analyses produced similar pooled estimates, with ORs ranging from 2.05 to 2.24 (all *p* < 0.05), supporting the stability and robustness of the findings.

### Results of the subgroup analysis

**Figure 3:**
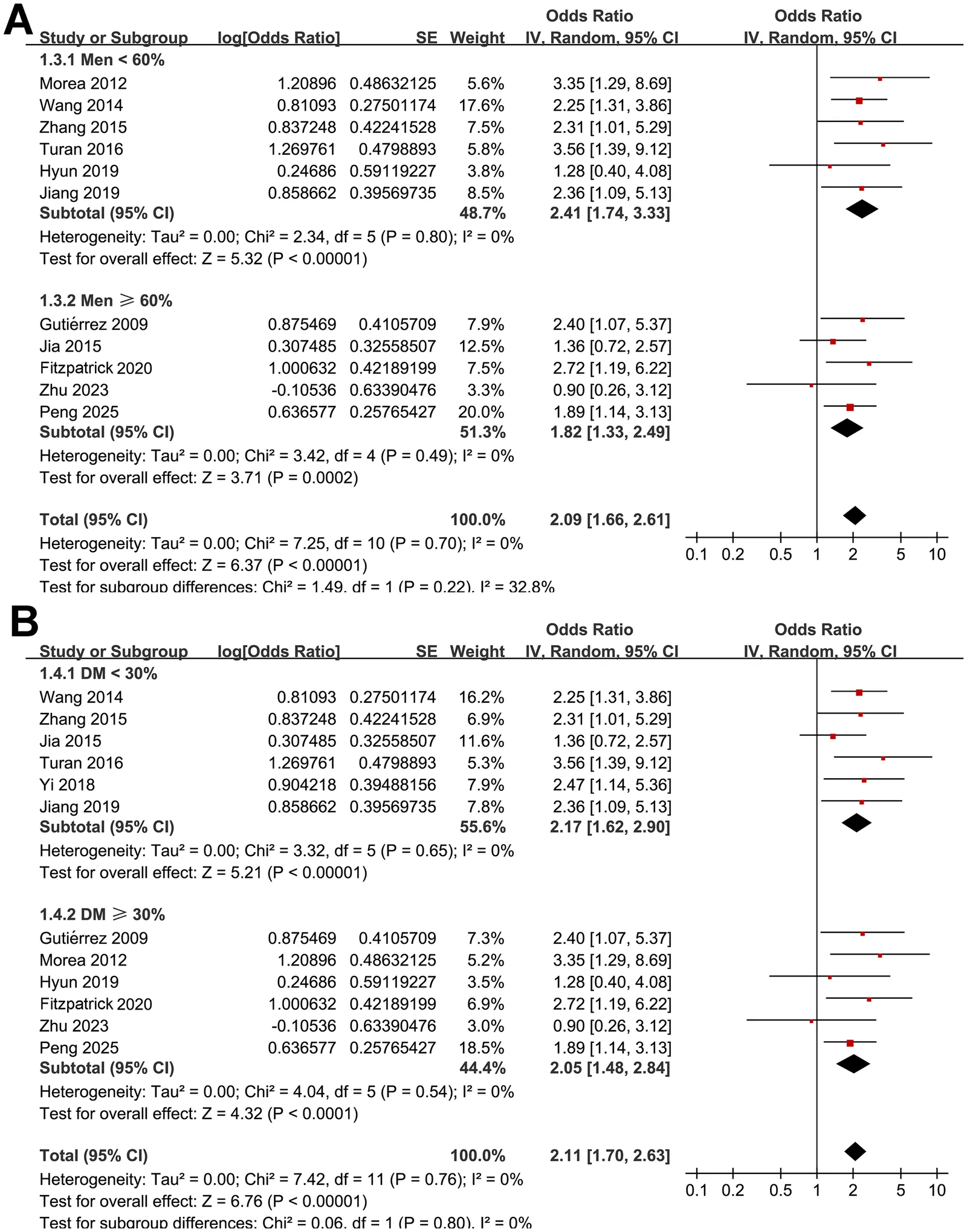
Forest plots showing the subgroup analysis of the association between blood FGF23 level and CAC in adult patients with CKD: **(A)** forest plots for the subgroup analysis according to the proportion of men of the studied population; and **(B)** forest plots for the subgroup analysis according to the proportion of diabetic patients of the studied population.

**Figure 4:**
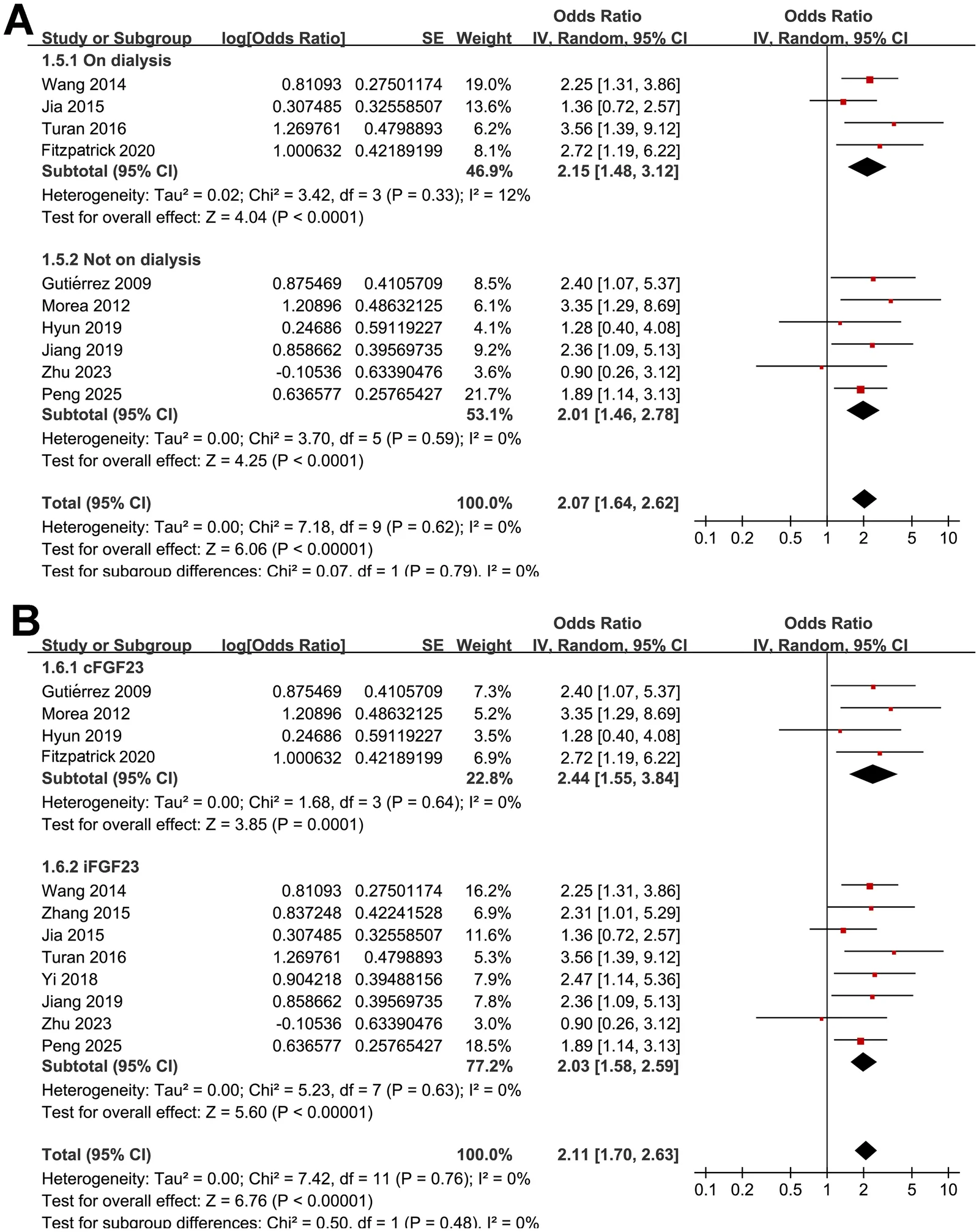
Forest plots showing the subgroup analysis of the association between blood FGF23 level and CAC in adult patients with CKD: **(A)** forest plots for the subgroup analysis in patients undergoing dialysis and those with predialysis CKD; and **(B)** forest plots for the subgroup analysis according to the type of FGF23 assay.

**Figure 5:**
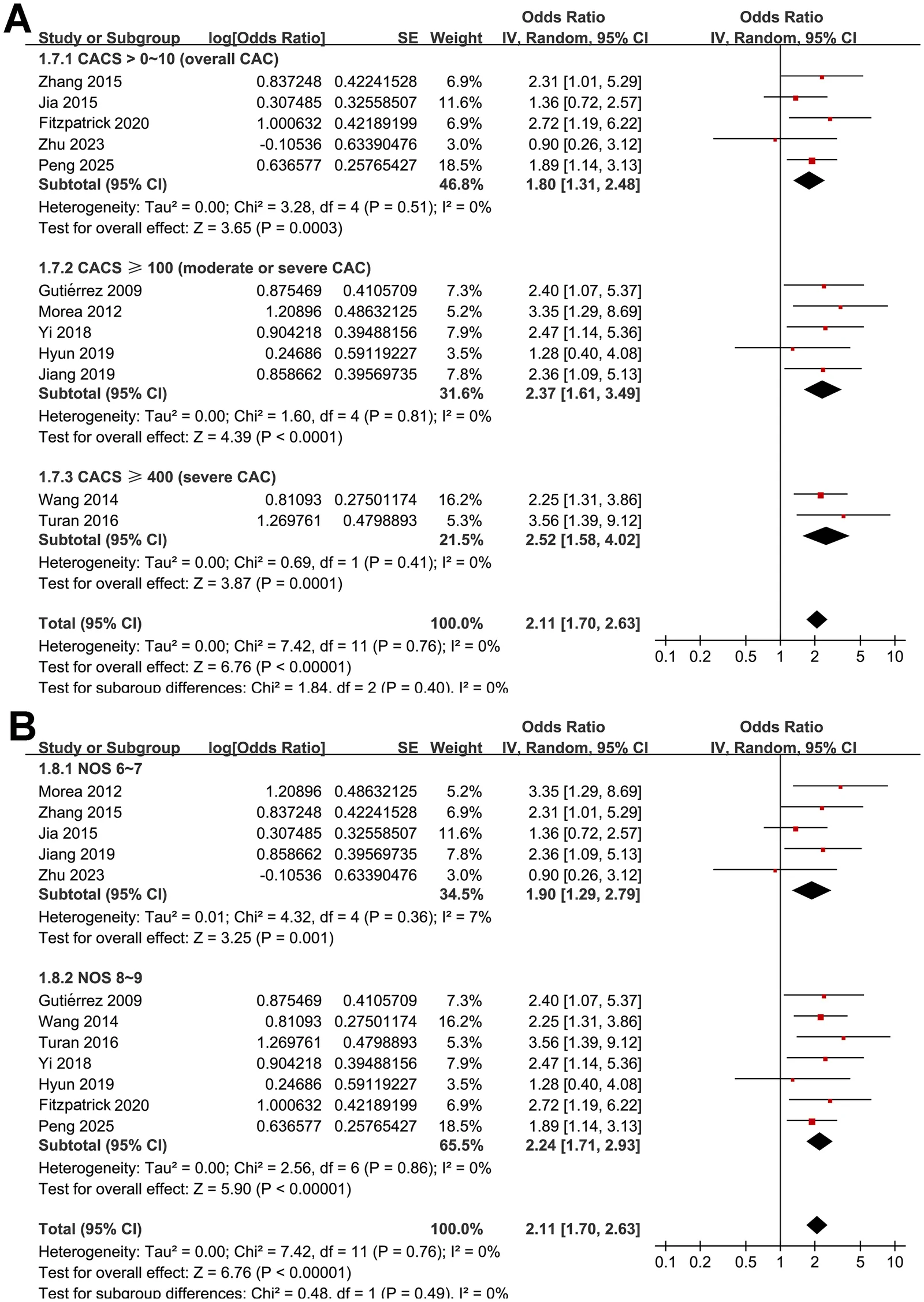
Forest plots showing the subgroup analysis of the association between blood FGF23 level and CAC in adult patients with CKD: **(A)** forest plots for the subgroup analysis according to the cutoff value of CACS; and **(B)** forest plots for the subgroup analysis according to the NOS scores.

Subgroup analyses demonstrated comparable associations between elevated circulating level of FGF23 and CAC among studies with the mean ages of the patients < 56 and ≥ 56 years (OR: 1.84 vs. 2.32, *p* for subgroup difference = 0.30; **Figure 2B**), between studies with the proportion of men < 60% and ≥ 60% (OR: 2.41 vs. 1.82, *p* for subgroup difference = 0.22; **Figure 3A**), and between studies with the proportion of patients with diabetes < 30% and ≥ 30% (OR: 2.17 vs. 2.05, *p* for subgroup difference = 0.80; **Figure 3B**). In addition, similar results were observed in studies including patients on dialysis and those with predialysis CKD (OR: 2.15 vs. 2.01, *p* for subgroup difference = 0.79; **Figure 4A**), and in studies measuring cFGF23 and iFGF23 (OR: 2.44 vs. 2.03, *p* for subgroup difference = 0.48; **Figure 4B**). Moreover, consistent associations were observed across subgroups defined by CACS thresholds, with pooled ORs of 1.80, 2.37, and 2.52 for studies using CACS > 0~10, CACS ≥ 100, and CACS ≥ 400, respectively. Although the magnitude of the association appeared to increase with CAC severity, the between-subgroup difference was not statistically significant (*p* for subgroup difference = 0.40; **Figure 5A**). Consistent results were also observed for studies with the NOS score of 6~7 and 8~9 (OR: 1.90 vs. 2.24, *p* for subgroup difference = 0.49; **Figure 5B**).

### Results of univariate meta-regression analyses

The results of the univariate meta-regression analysis are summarized in **Table 3**. None of the examined study-level characteristics, including sample size (*p* = 0.56), mean age (*p* = 0.61), proportion of men (*p* = 0.13), prevalence of diabetes (*p* = 0.99), CACS cutoff value (*p* = 0.30), or NOS score (*p* = 0.37), significantly influenced the association between circulating FGF23 levels and CAC in patients with CKD. These findings suggest that the observed association between FGF23 and CAC was generally consistent across studies with different demographic and methodological characteristics.

**Table 3:** Results of univariate meta-regression analysis.

Variables	OR for the association between blood FGF23 level and CAC in patients with CKD			
	Coefficient	95% CI	*p* values	Adjusted R^2^
Sample size	-0.00028	-0.00129 to 0.00074	0.56	0%
Mean age (years)	0.0072	-0.0232 to 0.0376	0.61	0%
Men (%)	-0.033	-0.078 to 0.012	0.13	0%
DM (%)	0.000083	-0.013482 to 0.013647	0.99	0%
Cutoff of CACS	0.00078	-0.00082 to 0.00238	0.30	0%
NOS	0.11	-0.15 to 0.37	0.37	0%

FGF23, fibroblast growth factor 23; CAC, coronary artery calcification; CKD, chronic kidney disease; OR, odds ratio; CI, confidence interval; DM, diabetes mellitus; CACS, coronary artery calcium score; NOS, Newcastle–Ottawa Scale.

### Evaluation of publication bias

**Figure 6:**
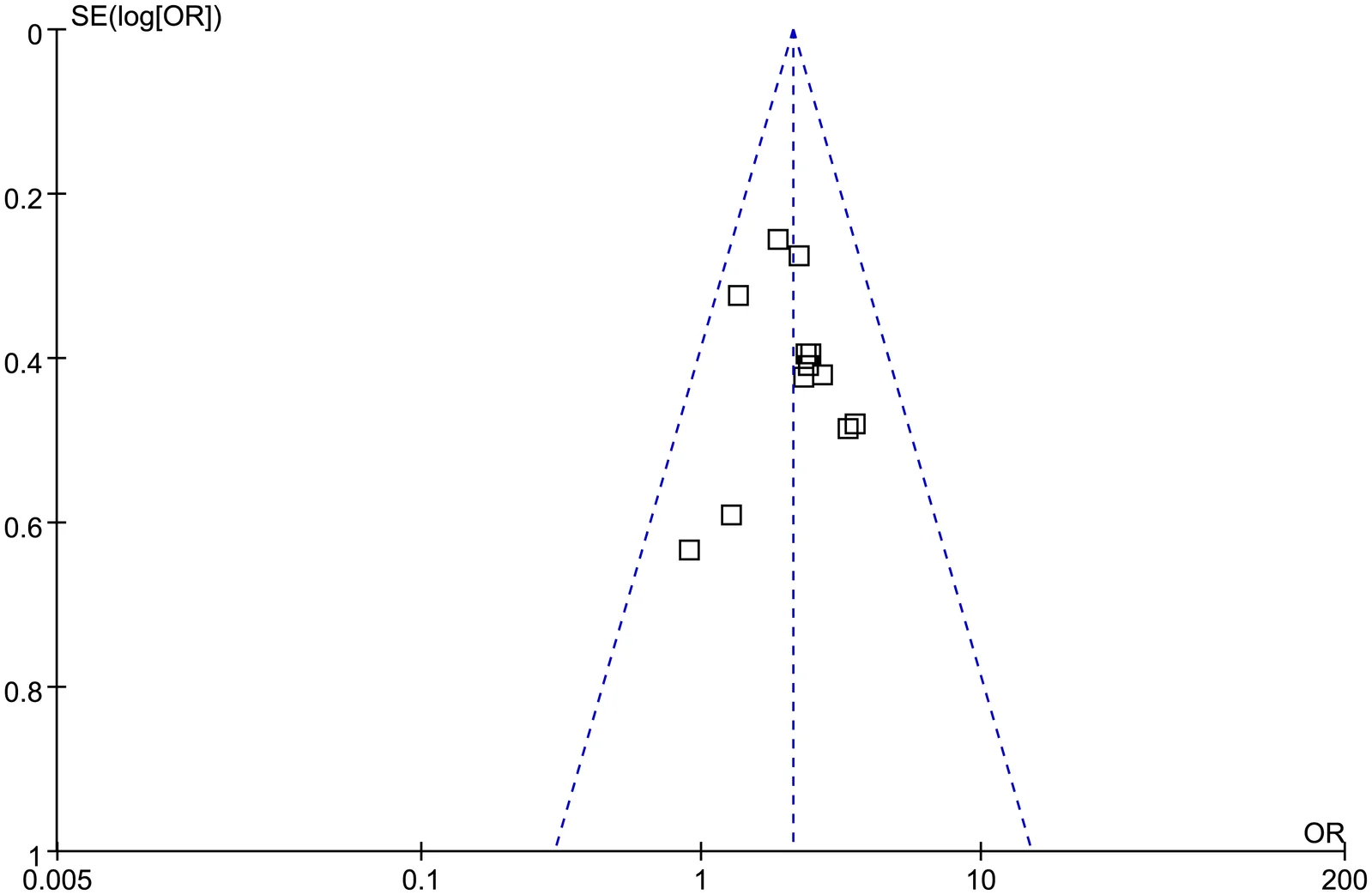
Funnel plots evaluating the publication bias for the meta-analysis of the association between blood FGF23 level and CAC in adult patients with CKD.

**Figure 6** shows that the funnel plots evaluating the association between circulating FGF23 levels and CAC in patients with CKD were broadly symmetrical. Similarly, Egger’s regression test did not reveal evidence of significant publication bias (*p* = 0.49). Nonetheless, caution is warranted when interpreting these findings given the relatively small number of included datasets (k = 12).

## Discussion

In this systematic review and meta-analysis of 12 cross-sectional studies involving 3, 466 patients with CKD, we found that elevated circulating FGF23 levels were independently associated with a higher prevalence of CAC. The association remained robust in sensitivity analyses and was generally consistent across subgroups stratified by demographic characteristics, dialysis status, FGF23 assay type, CAC severity, and study quality. Furthermore, none of the examined study-level characteristics significantly influenced the observed association in meta-regression analyses. Collectively, these findings support a close relationship between elevated FGF23 levels and CAC throughout the spectrum of CKD.

The mechanisms underlying the association between FGF23 and CAC are likely multifactorial. First, elevated FGF23 is a hallmark of CKD-MBD and primarily reflects phosphate retention resulting from declining kidney function (40, 41). Experimental studies have demonstrated that excess phosphate promotes osteogenic differentiation of VSMCs through upregulation of osteoblast-like transcription factors, leading to extracellular matrix mineralization and vascular calcification (42–44). Because FGF23 concentrations increase early during CKD progression in response to phosphate retention, elevated FGF23 may serve as a sensitive marker of the disordered mineral metabolism that drives vascular calcification (43). Second, accumulating evidence suggests that FGF23 may participate more directly in cardiovascular injury. Elevated FGF23 levels have been associated with endothelial dysfunction, increased arterial stiffness, oxidative stress, and chronic inflammation, all of which may facilitate vascular calcification (17, 45, 46). In addition, FGF23 may interact with abnormalities in calcium-phosphate homeostasis, parathyroid hormone signaling, and vitamin D metabolism, creating a pro-calcific milieu within the vascular wall (47). These mechanisms are biologically plausible because many of the pathways involved in CKD-MBD overlap with those implicated in vascular calcification and atherosclerosis.

Interestingly, the findings of our meta-analysis are further supported by several longitudinal studies evaluating CAC progression in CKD populations. In a prospective study of incident dialysis patients, Khan et al. (48) reported that higher baseline FGF23 levels were independently associated with accelerated CAC progression during follow-up, despite the absence of a significant association with baseline CAC severity. Similarly, Ozkok et al. (49) demonstrated that elevated serum FGF23 levels predicted CAC progression in maintenance hemodialysis patients and appeared particularly important during the early stages of the calcification process. More recently, analyses from the Chronic Renal Insufficiency Cohort (CRIC) study (50) identified FGF23 as an independent predictor of both incident CAC and CAC progression among patients with predialysis CKD. In addition, data from the China Dialysis Calcification Study (51) showed that higher FGF23 levels were independently associated with vascular calcification progression in dialysis populations. Although these studies were not eligible for quantitative synthesis because they defined CAC progression differently, their findings provide important longitudinal evidence supporting the biological relevance of FGF23 in vascular calcification.

Subgroup analyses further strengthened the robustness of our findings. The association between FGF23 and CAC was observed in both dialysis and predialysis CKD populations, suggesting that the relationship is not restricted to patients with advanced kidney failure. This observation is consistent with the progressive rise in FGF23 levels across CKD stages and suggests that FGF23-associated vascular injury may begin before the initiation of dialysis (52). Similarly, comparable associations were observed for studies measuring cFGF23 and iFGF23. Because cFGF23 assays detect both intact hormone and cleavage fragments whereas iFGF23 assays specifically measure biologically active FGF23, this finding suggests that the association is not dependent on the assay methodology used. We also observed generally consistent associations across studies with different proportions of male participants, mean ages, diabetes prevalence, and methodological quality, indicating that the observed relationship is relatively stable across diverse patient populations. Another notable finding was that the association appeared numerically stronger in studies evaluating more advanced CAC severity, although the between-subgroup difference was not statistically significant. This pattern may suggest that FGF23 becomes increasingly relevant as vascular calcification progresses, which is biologically plausible given the cumulative effects of long-standing phosphate dysregulation and CKD-MBD. However, because the subgroup differences did not reach statistical significance and the number of studies in some categories was limited, this observation should be interpreted cautiously. Consistently, meta-regression analyses did not identify significant effects of sample size, age, sex distribution, diabetes prevalence, CAC threshold, or NOS score on the pooled effect estimate. While these findings support the consistency of the association across different study settings, the absence of significant moderators should not be interpreted as definitive evidence that these factors are unimportant. Meta-regression analyses are inherently limited by the relatively small number of available studies and the use of study-level rather than individual-level data.

## Strengths and limitations

This meta-analysis has several strengths. To our knowledge, this is the first study to quantitatively synthesize the association between circulating FGF23 levels and CAC in patients with CKD. We performed a comprehensive and up-to-date literature search across both English and Chinese databases, thereby minimizing the likelihood of missing relevant studies. All included studies reported adjusted effect estimates, allowing the pooled analysis to account for important confounding factors. In addition, we performed extensive sensitivity analyses, multiple prespecified subgroup analyses, and meta-regression analyses, all of which supported the robustness and consistency of the findings. The absence of significant statistical heterogeneity further strengthens confidence in the pooled results.

Several limitations should also be acknowledged. First, all included studies adopted cross-sectional designs. Therefore, the temporal relationship between elevated FGF23 levels and CAC cannot be established, and causality cannot be inferred. It remains possible that elevated FGF23 represents a consequence rather than a cause of the pathological processes associated with vascular calcification. Second, although statistical heterogeneity was negligible, important clinical heterogeneity remained. The included studies enrolled diverse CKD populations, including predialysis CKD, maintenance hemodialysis, peritoneal dialysis, and mixed cohorts, with some variation in CKD diagnostic criteria and staging definitions. Moreover, predialysis populations ranged from early-stage CKD to advanced stage 5 diseases not requiring dialysis. Because most studies did not report stage-specific effect estimates, more detailed analyses according to CKD stage were not feasible. Therefore, the consistent associations observed across dialysis and predialysis subgroups should be interpreted cautiously, and potential differences across individual CKD stages cannot be excluded. Third, considerable variability existed in the measurement of FGF23, including the use of cFGF23 and iFGF23 assays, different biological specimens, and different cutoff values. Fourth, CAC was defined using different thresholds across studies, ranging from the presence of any detectable calcification to severe calcification. Fifth, the adjustment models varied substantially among studies, and residual confounding from unmeasured factors such as dietary phosphate intake, medication use, genetic susceptibility, inflammation, or other CKD-MBD biomarkers cannot be excluded. Finally, because individual participant data were unavailable, more detailed analyses according to CKD stage, dialysis modality, phosphate levels, or other clinically relevant factors could not be performed.

### Clinical implications and future direction

The clinical implications of these findings deserve consideration. Given the strong and consistent association observed across studies, circulating FGF23 may represent a useful biomarker for identifying patients with CKD who are at increased risk of CAC. However, because the available evidence is predominantly cross-sectional, our findings should not be interpreted as evidence that lowering FGF23 levels will prevent or reverse vascular calcification. Future prospective cohort studies should clarify the temporal relationship between FGF23 and CAC development, while mechanistic studies are needed to determine whether FGF23 directly contributes to vascular calcification or primarily reflects underlying disturbances in mineral metabolism. Interventional studies targeting phosphate balance, CKD-MBD, or FGF23-related pathways may further help determine whether modification of FGF23 can translate into cardiovascular benefit.

## Conclusions

In conclusion, this meta-analysis demonstrates that elevated circulating FGF23 levels are associated with a higher prevalence of CAC in patients with CKD. The association was consistent across multiple clinically relevant subgroups and was supported by available longitudinal studies linking FGF23 to CAC progression. Although these findings suggest that elevated FGF23 levels may serve as a potential biomarker associated with vascular calcification in CKD, prospective and mechanistic studies are required to clarify causality and determine its clinical utility in cardiovascular risk stratification and therapeutic decision-making.
