# Supplementary material for: Association between fibroblast growth factor 23 and coronary artery calcification in patients with chronic kidney disease: a meta-analysis
Source: Front Endocrinol (Lausanne). 2026 Sep 7;17:1900670. doi: 10.3389/fendo.2026.1900670 (PMC13590301; doi:10.3389/fendo.2026.1900670)
Supplement: Supplementary file 1 [file Table1.docx]

**Detailed search strategy for each database**

**PubMed**

("Fibroblast Growth Factor 23"[Mesh] OR "fibroblast growth factor 23"[tiab] OR FGF23[tiab] OR FGF-23[tiab] OR "FGF 23"[tiab]) AND ("Coronary Artery Calcification"[Mesh] OR "coronary artery calcification"[tiab] OR "coronary calcification"[tiab] OR CAC[tiab] OR CACS[tiab] OR "coronary artery calcium"[tiab] OR "coronary artery calcium score"[tiab] OR Agatston[tiab]) AND ("Renal Insufficiency, Chronic"[Mesh] OR "Kidney Failure, Chronic"[Mesh] OR "Renal Dialysis"[Mesh] OR "chronic kidney disease"[tiab] OR CKD[tiab] OR "chronic renal insufficiency"[tiab] OR "chronic renal failure"[tiab] OR "renal insufficiency"[tiab] OR "kidney failure"[tiab] OR "end-stage renal disease"[tiab] OR ESRD[tiab] OR dialysis[tiab] OR hemodialysis[tiab] OR haemodialysis[tiab] OR "peritoneal dialysis"[tiab])

**Embase**

('fibroblast growth factor 23'/exp OR 'fibroblast growth factor 23':ti,ab OR fgf23:ti,ab OR fgf-23:ti,ab OR 'fgf 23':ti,ab) AND ('coronary artery calcification'/exp OR 'coronary artery calcium score'/exp OR 'coronary artery calcification':ti,ab OR 'coronary calcification':ti,ab OR cac:ti,ab OR cacs:ti,ab OR 'coronary artery calcium':ti,ab OR 'coronary artery calcium score':ti,ab OR agatston:ti,ab) AND ('chronic kidney disease'/exp OR 'chronic kidney failure'/exp OR 'end stage renal disease'/exp OR 'dialysis'/exp OR 'hemodialysis'/exp OR 'peritoneal dialysis'/exp OR 'chronic kidney disease':ti,ab OR ckd:ti,ab OR 'chronic renal insufficiency':ti,ab OR 'chronic renal failure':ti,ab OR 'renal insufficiency':ti,ab OR 'kidney failure':ti,ab OR 'end-stage renal disease':ti,ab OR esrd:ti,ab OR dialysis:ti,ab OR hemodialysis:ti,ab OR haemodialysis:ti,ab OR 'peritoneal dialysis':ti,ab)

**Web of Science**

TS=(("fibroblast growth factor 23" OR FGF23 OR FGF-23 OR "FGF 23") AND ("coronary artery calcification" OR "coronary calcification" OR CAC OR CACS OR "coronary artery calcium" OR "coronary artery calcium score" OR Agatston) AND ("chronic kidney disease" OR CKD OR "chronic renal insufficiency" OR "chronic renal failure" OR "renal insufficiency" OR "kidney failure" OR "end-stage renal disease" OR ESRD OR dialysis OR hemodialysis OR haemodialysis OR "peritoneal dialysis"))

**Wanfang**

主题: (成纤维细胞生长因子23 OR FGF23 OR FGF-23) AND (冠状动脉钙化 OR 冠脉钙化 OR 冠状动脉钙积分 OR 冠状动脉钙化积分 OR CAC OR CACS) AND (慢性肾脏病 OR 慢性肾病 OR CKD OR 慢性肾功能不全 OR 慢性肾衰竭 OR 终末期肾病 OR ESRD OR 透析 OR 血液透析 OR 腹膜透析)

**CNKI**

SU=(成纤维细胞生长因子23 OR FGF23 OR FGF-23) AND (冠状动脉钙化 OR 冠脉钙化 OR 冠状动脉钙积分 OR 冠状动脉钙化积分 OR CAC OR CACS) AND (慢性肾脏病 OR 慢性肾病 OR CKD OR 慢性肾功能不全 OR 慢性肾衰竭 OR 终末期肾病 OR ESRD OR 透析 OR 血液透析 OR 腹膜透析)
